# A Comparative Study on the Mechanical Properties of Open-Hole Carbon Fiber-Reinforced Thermoplastic and Thermosetting Composite Materials

**DOI:** 10.3390/polym15224468

**Published:** 2023-11-20

**Authors:** Liu Han, Yao Song, Hui Qi, Jin-Shui Yang, Shuang Li, Ping-An Liu

**Affiliations:** 1 College of Aerospace and Civil Engineering, Harbin Engineering University, Harbin 150001, China; yangjinshui@hrbeu.edu.cn (J.-S.Y.); liupingan@hrbeu.edu.cn (P.-A.L.); 2 AVIC Harbin Aircraft Industry Croup Co., Ltd., Harbin 150066, China; songy056@avic.com; 3 Qingdao Innovation and Development Base, Harbin Engineering University, Qingdao 266000, China; lishuang@hrbeu.edu.cn

**Keywords:** thermoplastic composites, thermosetting composites, open-hole, macroscopic damage, micro-failure modes, CDM

## Abstract

In this paper, the damage initiation/propagation mechanisms and failure modes of open-hole carbon fiber-reinforced thermoplastic composites and thermosetting composites with tension, compression, and bearing loads are investigated, respectively, by experiments and finite element simulations. The experimental evaluations are performed on the specimens using the Combined Loading Compression (CLC) test method, the tensile test method, and the single-shear test method. The differences in macroscopic damage initiation, evolution mode, and damage characteristics between thermoplastic composite materials and thermosetting composite material open-hole structures are obtained and analyzed under compressive load. Based on scanning electron microscope SEM images, a comparative analysis is conducted on the micro-failure modes of fibers, matrices, and fiber/matrix interfaces in the open-hole structures of thermoplastic and thermosetting composites under compressive load. The differences between thermoplastic and thermosetting composites were analyzed from the micro-failure mechanism. Finally, based on continuum damage mechanics (CDM), a damage model is also developed for predicting the initiation and propagation of damage in thermoplastic composites. The model, which can capture fiber breakage and matrix crack, as well as the nonlinear response, is used to conduct virtual compression tests, tensile test, and single-shear test, respectively. Numerical simulation results are compared with the extracted experimental results. The displacement-load curve and failure modes match the experimental result, which indicates that the finite element model has good reliability.

## 1. Introduction

As an ideal lightweight material, thermoplastic composites are widely used in aerospace, automotive, wind turbine blades, and other fields [1]. To meet the requirements of damage tolerance, long life, seaworthiness, and environmental protection of aircraft structures, it is the most effective way to pursue structural effects by using thermoplastic composites with high damage resistance, fatigue resistance, easy repairability and recyclability [2,3]. High-performance thermoplastic composites usually refer to high-strength glass fiber, aramid fiber, and carbon fiber-reinforced thermoplastic resin composites. The high-performance resins commonly used in aerospace are polyetherimide (PEI), polyphenylene sulfide (PPS), polyetheretherketone (PEEK), polyetherketoneketone (PEKK), etc. [4].

Due to the requirements of assembly or structural function, there are often holes or openings in the aviation structure, which makes the open-hole composite component become a typical structure on the aircraft [5]. Since the fibers around the hole are cut off, the stress concentration problem at the hole edge is very prominent. It causes the coupling of multiple damage modes, which, in turn, leads to catastrophic damage [6,7]. Therefore, the open-hole composite structure is widely used in the determination of allowable value and the evaluation of damage tolerance performance.

The tensile, compressive, and bearing properties of open-hole thermoplastic composites are the basic mechanical characteristics. In recent decades, scholars have conducted experimental research on the damage modes, failure mechanisms, and failure strength of open-hole thermoplastic composite structures. However, there are few comparisons with the open-hole thermosetting composite structure. Saha et al. [8] obtained the strain distribution and strain concentration coefficient at the hole edge by arranging strain gauges inside and around the hole. It was found that the stress concentration and delamination damage appeared first at the hole edge. With the continuous expansion of delamination, fiber fracture and matrix shear cracking were induced. Boey and Kwon [9] studied the compression damage process of notched woven fabric composites with different strain rates. The results show that the bearing capacity of the specimen increases with an increase in strain rate. During the loading process, the matrix cracking and fiber micro-buckling first occurred at the hole edge. With the increase in load, the local damage area at the hole edge continued to expand. Finally, the composite material was damaged instantaneously. Green et al. [10,11] explored the tensile ultimate strength and failure mode of composite open-hole plates under quasi-isotropic ply. The effects of different pore sizes and plies were analyzed. The results show that the matrix crack and local micro-delamination are first generated at the hole edge. Then, the delamination penetrates the width direction and thickness direction of the laminate along the matrix crack, resulting in a decrease in stiffness and delamination failure. Wisnom et al. [12] established the variation law of laminate failure load by the open-hole tensile test of quasi-isotropic laminates on the basis of Green’s research. The influence of delamination on the open-hole tensile ultimate strength and failure mode was analyzed. In addition, the influence of hole size on the failure mode of the notched area was discussed. O’Higgins et al. [13] used high-strength glass fiber composites and carbon fiber composites to make open-hole laminate, respectively, and studied the tensile failure process and damage evolution of the two material laminates. Wang et al. [14] proposed a semicircular bearing strength test method based on the single-shear bearing strength test of carbon fiber composite laminates. This method can obtain the mechanism of bearing failure and analyze the relationship between bearing failure and pre-tightening force, lateral restraint, and stacking sequence. The results show that lateral restraint can restrain the propagation of shear cracks and change compressive failure from brittle to progressive failure. Heimbs [15] compared the failure and strain rate of carbon fiber composite laminates with countersunk bolt connections under static and dynamic loads (10 m/s). Under high-speed load, the failure mode of a single-shear connection changes due to its good energy absorption characteristics.

Therefore, the tension, compression, and bearing properties of the open-hole carbon fiber (CF)-reinforced thermoplastic composite laminate are investigated by experiments and simulations in this paper. The failure modes and mechanisms are analyzed via the finite element method. Furthermore, a comparison with open-hole thermosetting composite laminate is made. Some meaningful conclusions are obtained, which are of great significance to optimizing the structural design and further expanding the application of thermoplastic composites.

## 2. Constitutive Model and Experiment

### 2.1. Material and Samples

**Figure 1:**
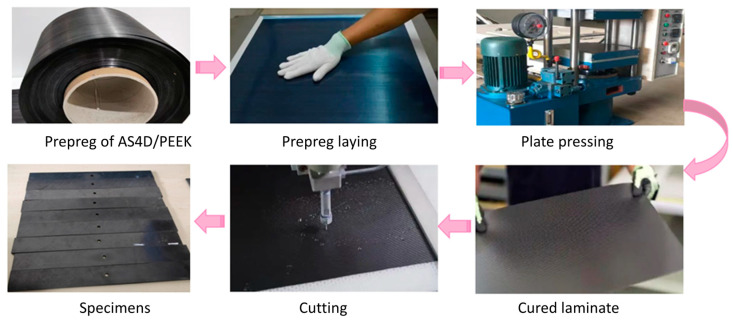
Manufacture of test samples.

The open-hole carbon fiber-reinforced thermoplastic composite specimens are made of AS4D/PEEK prepreg, which is provided by TenCate company in Almelo, The Netherlands. The thermoplastic composite laminates are stacked with [45/0/–45/90/45/0/–45/90/45/0/–45/90/45/0/–45/90]s and manufactured by the hot-pressing process. Firstly, the temperature is raised to 385 °C. Then 0.7 MPa pressure is applied and held for 30 min. Finally, the laminates are cooled to room temperature (the cooling rate is 4 °C/min). The prepreg and laminates are shown in Figure 1. As shown in Figure 1, the laminates are cut by a water-cooled diamond cutter to avoid matrix carbonization. The specimens are cut and assembled according to standards ASTM D6484 [16], ASTM D5766 [17], and ASTM D5961 [18] for compression, stretching, and bearing tests. The thermosetting composite specimens for comparison are manufactured by vacuum hot pressing. The material is carbon fiber-reinforced thermosetting composites CCF300/Epoxy. The mechanical properties of CCF300/Epoxy prepreg are listed in Table 1. The mechanical properties of AS4D/PEEK prepreg are shown in Table 2, and Table 3 shows the properties of AS4D and CCF300 carbon fibers. It is shown that these two fibers have similar characteristics. Table 4 shows the properties of the PEEK and CCF300 matrixes. This article mainly studies the effects of thermoplastic and thermosetting resins on the opening and impact properties of composite materials.

### 2.2. Experimental

All of the tests are performed on ±100 kN electronic tensile testing machine INSTRON 1195. The ASTM D6484 is referenced to compression tests [16]. The anti-instability devices are applied to prevent the lateral instability of the specimens. The compression speed is 2 mm/min. The test is stopped when the maximum load drops to 30%. The tensile test is carried out according to ASTM D5766 [17]. The specimens are continuously loaded with 2 mm/min until they are destroyed.

The compression and tensile strengths are

(1) $$\sigma =\frac{P_{\max}}{wt}$$

where $P_{\max}$ is the failure load; w is the average width, and t is the average thickness. Considering the difference in fiber content among specimens, the strength is expressed as

(2) $$\sigma_{0}=\sigma \times \frac{t_{0}}{t}$$

where $\sigma_{0}$ is the regularization strength; σ is the measured strength, t is the nominal thickness, and $t_{0}$ is the measured thickness. 

Method B of ASTM D5961 is used for bearing strength tests [18]. Anti-instability devices are also applied to prevent lateral instability. The loading speed is 2 mm/min. The tests are stopped until the load no longer increases. The bearing strength is

(3) $$\sigma_{br}=\frac{P_{\max}}{kDh}$$

where $\sigma_{br}$ is the ultimate bearing strength; $P_{\max}$ is the maximum load; k is the number of fasteners; D is the diameter of the hole, and h is the average thickness of the specimens.

### 2.3. Simulation

#### 2.3.1. Damage Constitutive

The CF/PEEK composite is an orthotropic material. In the local Cartesian coordinate system, if the fundamental vector corresponds to the warp and weft directions of orthogonal composite materials, the material is symmetric with 1–3 and 2–3 planes, and it is an orthogonal anisotropic material with its stress–strain relationship. The three-dimensional stress–strain relationship of a single-layer plate can be expressed as follows [19]:

(4) $$\left[\begin{array}{c} \sigma_{11}\\ \sigma_{22}\\ \sigma_{33}\\ \tau_{23}\\ \tau_{31}\\ \tau_{12} \end{array}\right]=\left[\begin{array}{cccccc} C_{11}{}^{0} & C_{12}{}^{0} & C_{13}{}^{0} & 0 & 0 & 0\\ C_{12}{}^{0} & C_{22}{}^{0} & C_{23}{}^{0} & 0 & 0 & 0\\ C_{13}{}^{0} & C_{23}{}^{0} & C_{33}{}^{0} & 0 & 0 & 0\\ 0 & 0 & 0 & C_{44}{}^{0} & 0 & 0\\ 0 & 0 & 0 & 0 & C_{55}{}^{0} & 0\\ 0 & 0 & 0 & 0 & 0 & C_{66}{}^{0} \end{array}\right]\left[\begin{array}{c} \varepsilon_{11}\\ \varepsilon_{22}\\ \varepsilon_{33}\\ \gamma_{23}\\ \gamma_{31}\\ \gamma_{12} \end{array}\right]$$

where $\sigma_{ij}(i,j=1,2,3)$ is the stress components in each direction; $\varepsilon_{ij}(i,j=1,2,3)$ is the strain components in each direction, and $C_{ij}{}^{0}(i,j=1,2,3,4,5,6)$ is the undamaged stiffness coefficient. The subscript 1 indicates the direction parallel to the fiber; 2 indicates the direction perpendicular to the fiber, and 3 indicates the direction of laminate thickness.

According to the relevant knowledge of composite strength of materials, the engineering constant expression of the undamaged stiffness matrix C is

(5) $$C=\frac{1}{\Omega }\left[\begin{array}{cccccc} E_{1}{}^{0}\left(1-v_{23}v_{32}\right) & E_{2}{}^{0}\left(v_{12}+v_{32}v_{13}\right) & E_{3}{}^{0}\left(v_{13}+v_{12}v_{23}\right) & 0 & 0 & 0\\ E_{1}{}^{0}\left(v_{21}+v_{31}v_{23}\right) & E_{2}{}^{0}\left(1-v_{13}v_{31}\right) & E_{3}{}^{0}\left(v_{23}+v_{21}v_{13}\right) & 0 & 0 & 0\\ E_{1}{}^{0}\left(v_{31}+v_{21}v_{32}\right) & E_{2}{}^{0}\left(v_{32}+v_{12}v_{31}\right) & E_{3}{}^{0}\left(1-v_{12}v_{21}\right) & 0 & 0 & 0\\ 0 & 0 & 0 & \Omega G_{23}{}^{0} & 0 & 0\\ 0 & 0 & 0 & 0 & \Omega G_{31}{}^{0} & 0\\ 0 & 0 & 0 & 0 & 0 & \Omega G_{12}{}^{0} \end{array}\right]$$

where $\Omega =1-v_{12}v_{21}-v_{23}v_{32}-v_{31}v_{13}-2v_{12}v_{23}v_{31}$ and $E_{1}{}^{0}\;E_{2}{}^{0}\;E_{3}{}^{0}$ are the $\sigma_{ij}(i,j=1,2,3)$, the elastic modulus of undamaged single-layer plate in three directions; $v_{ij}(i\neq j,i,j=1,2,3)$ are the Poisson’s ratios, and $G_{ij}{}^{0}(i,j=1,2,3)$ are the Shear modulus. The elastic modulus and Poisson’s ratio meet the following relationship:

(6) $$\frac{E_{i}}{E_{j}}=\frac{v_{ji}}{v_{ij}}$$

Under small deformation conditions, the CF/PEEK composite material still meets the anisotropic condition after being damaged. According to the theory of CDM, when damage occurs, the stiffness of the laminate is lower than that of the undamaged laminate, and the stiffness degradation caused by damage can be represented by stiffness reduction, then the elastic stress–strain relationship with damage is as follows [20,21]:

(7) $$\sigma =C_{d}\varepsilon^{el}$$

(8) $$C_{d}=CM$$

where M is the damage tensor for thermoplastic composites, in which fiber and matrix damage the parameters. $C_{d}$ is the damage stiffness matrix; $\varepsilon^{el}$ is the elastic strain.

(9) $$M=\left[\begin{array}{cccccc} 1-d_{11} & 0 & 0 & 0 & 0 & 0\\ 0 & 1-d_{22} & 0 & 0 & 0 & 0\\ 0 & 0 & 1-d_{33} & 0 & 0 & 0\\ 0 & 0 & 0 & 1-d_{23} & 0 & 0\\ 0 & 0 & 0 & 0 & 1-d_{31} & 0\\ 0 & 0 & 0 & 0 & 0 & 1-d_{12} \end{array}\right]\;\begin{array}{l} d_{11}=1-\left(1-d_{11}^{T}\right)\left(1-d_{11}^{C}\right)\\ d_{22}=1-\left(1-d_{22}^{T}\right)\left(1-d_{22}^{C}\right)\\ d_{33}=1-\left(1-d_{22}^{T}\right)\left(1-d_{22}^{C}\right)\\ d_{23}=1-\left(1-d_{11}^{T}\right)\left(1-d_{11}^{C}\right)\left(1-d_{22}^{T}\right)\left(1-d_{22}^{C}\right)\\ d_{31}=1-\left(1-d_{11}^{T}\right)\left(1-d_{11}^{C}\right)\left(1-d_{22}^{T}\right)\left(1-d_{22}^{C}\right)\\ d_{12}=1-\left(1-d_{11}^{T}\right)\left(1-d_{11}^{C}\right)\left(1-d_{22}^{T}\right)\left(1-d_{22}^{C}\right) \end{array}$$

where *d_ij_* is the damage factor; $d_{11}^{T},\;d_{11}^{C},\;d_{22}^{T},\;d_{22}^{C}$ are the damage factors of longitudinal tension, longitudinal compression, transverse tension, and transverse compression.

#### 2.3.2. Damage Initiation Criteria

The failure mechanism of composite materials is complex, and the Hashin failure criterion is the most commonly used in failure judgment [22]. The failure of materials is represented by the failure factor *FI*, and the corresponding failure criteria are shown as follows:

Fiber tensile damage $\varepsilon_{11}\geq 0$

(10) $$\phi_{1+}={\left(\frac{\varepsilon_{11}}{\varepsilon_{11}^{T}}\right)}^{2}+\frac{1}{\varepsilon_{12}^{2}}\left(\varepsilon_{12}^{2}+\varepsilon_{13}^{2}\right)$$

Fiber compression damage $\varepsilon_{11}<0$

(11) $$\phi_{1-}={\left(\frac{\varepsilon_{11}}{\varepsilon_{11}^{c}}\right)}^{2}$$

Matrix tensile damage $\varepsilon_{22}+\varepsilon_{33}\geq 0$

(12) $$\phi_{2+}={\left(\frac{\varepsilon_{22}+\varepsilon_{33}}{\varepsilon_{22}^{T}}\right)}^{2}+\frac{1}{\varepsilon_{23}^{2}}\left(\varepsilon_{23}^{2}-\varepsilon_{22}\varepsilon_{33}\right)+\frac{1}{\varepsilon_{12}^{2}}\left(\varepsilon_{12}^{2}+\varepsilon_{13}^{2}\right)$$

Matrix compression damage $\varepsilon_{22}+\varepsilon_{33}<0$

(13) $$\begin{array}{l} \phi_{2-}={\left(\frac{\varepsilon_{22}+\varepsilon_{33}}{2\varepsilon_{23}{}^{S}}\right)}^{2}+\left(\frac{\varepsilon_{22}+\varepsilon_{33}}{\varepsilon_{22}^{C}}\right)\left[{\left(\frac{\varepsilon_{22}^{c}}{2\varepsilon_{23}{}^{S}}\right)}^{2}-1\right]+\\ \frac{1}{\varepsilon_{23}^{2}}\left(\varepsilon_{23}^{2}-\varepsilon_{22}\varepsilon_{33}\right)+\frac{1}{\varepsilon_{12}^{2}}\left(\varepsilon_{12}^{2}+\varepsilon_{13}^{2}\right) \end{array}$$

where $\phi_{\alpha }$(α=1±,2±) is the tension/compressive failure factors in fiber and matrix directions, and $\phi_{\alpha }>1$ indicates the occurrence of initial damage. $\varepsilon_{11}$, $\varepsilon_{22}$, $\varepsilon_{12}\;$ is the longitudinal and transverse tensile/compressive and shear strains. $\varepsilon_{i,1}^{T},\varepsilon_{i,1}^{C},\;\varepsilon_{i,2}^{T},\;\varepsilon_{i,2}^{C}$ are the initial damage strains of longitudinal tension, longitudinal compression, transverse tension, and transverse compression. $\varepsilon_{i,12},\;\varepsilon_{i,23}$ are the shear initial failure strains. $\varepsilon_{11}^{T}=X_{T}/E_{1}{}^{0}$, $\varepsilon_{11}^{C}=X_{C}/E_{1}{}^{0}$, $\varepsilon_{22}^{T}=Y_{T}/E_{2}{}^{0}$, $\varepsilon_{22}^{C}=Y_{C}/E_{2}{}^{0}$, $\varepsilon_{12}=S_{12}/\left(2G_{12}{}^{0}\right)$, $\varepsilon_{13}=S_{13}/\left(2G_{13}{}^{0}\right)$, $\varepsilon_{23}=S_{23}/\left(2G_{23}{}^{0}\right)$.

#### 2.3.3. Damage Propagation

After the material meets the above strain-based 3D Hashin failure criterion at a certain integral point, the initial failure occurs, the stiffness does not immediately decrease to 0, and the mechanical properties of the material need to be degraded. The damaged evolution of composites is a process of energy release. In the process of strain energy release, the materials in the damage area will appear to be a “softening” phenomenon, which is manifested in the matrix micro-cracks, and the separations of fibers and matrix occur at the interface, fiber fracture, etc. Macroscopic manifestations include the degradation of stiffness and the decline in the macro ultimate strength value [23]. Progressive failure analysis of composite materials is usually expressed by the stiffness degradation of materials. This analysis uses the method in reference to exponential decay attenuating the damage variable $d_{\alpha }$ of tensile/compression failure of materials based on the fracture toughness of materials (α=±1,±2) [24,25,26,27,28].

(14) $$d_{\alpha }=1-\frac{1}{r_{\alpha }}\exp\left[-\frac{2g_{0}^{\alpha }L_{c}}{G_{f}^{\alpha }-g_{0}^{\alpha }L_{c}}\left(r_{\alpha }-1\right)\right]$$

where $d_{\alpha }$ is the damage variable. $r_{\alpha }$ is the damage threshold, which is initially uniformly set, and after the onset of damage, ($\phi_{i+}=1\mathrm{or}\phi_{i-}=1$). $r_{i+}(t)=\max_{\tau \leq t}\phi_{i+}(\tau )$, $r_{i-}(t)=\max_{\tau \leq t}\phi_{i-}(\tau )$. The damage threshold $r_{\alpha }$ is monotonically increasing ${\dot{r}}_{\alpha }(t)⩾0\;(\alpha =\pm 1,\pm 2)$. $G_{f}^{\alpha }$ is the unit area fracture energy of materials under uniaxial tension and compression in directions 1 and 2. $L_{c}$ is the element characteristic length, used for the dependence of the continuum damage mechanics model on element size. $g_{0}^{\alpha }$ is the unit volume fracture energy density (elastic energy density) at the initiation point of tensile and compressive damage in materials’ directions 1 and 2.

(15) $$g_{0}^{\alpha }=\frac{\left(X_{i}^{\alpha }\right)}{2E_{i}^{\alpha }},(\alpha =\pm 1,\pm 2)$$

where $E_{i}^{a}$ is the uniaxial tensile and compressive modulus of materials in direction 1 and direction 2.

#### 2.3.4. Elastic–Plastic Shear Failure Criterion

**Figure 2:**
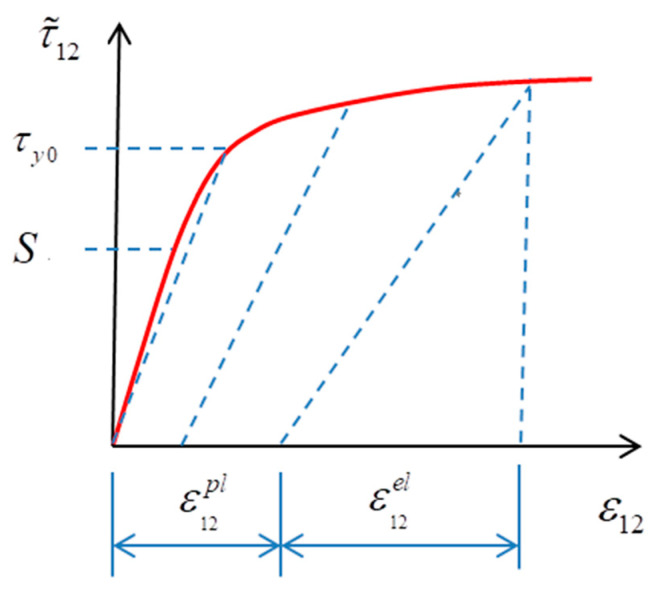
Typical shear stress–strain curve of composite materials [28].

The elastic damage model is suitable for fiber-dominated failure modes. For in-plane shear, the deformation is controlled by the behavior of the matrix. What is more, the nonlinear behavior of the shear stress–strain curve (shown in Figure 2) is obvious under the shear load [28].

Considering shear nonlinear deformation and according to the small strain assumption, $\varepsilon_{11}^{pl}=\varepsilon_{22}^{pl}=0,\;\varepsilon_{12}^{pl}\neq 0$, so the total shear strain is

(16) $$\varepsilon_{12}=\varepsilon_{12}^{el}+\varepsilon_{12}^{pl}$$

After the initial shear damage occurs, the equivalent shear stress and equivalent shear plastic strain are

(17) $${\tilde{\tau }}_{12}=\frac{\tau_{12}}{1-d_{12}}=2G_{12}\varepsilon_{12}{}^{el}=2G_{12}\left(\varepsilon_{12}-\varepsilon_{12}{}^{pl}\right)$$

(18) $${\tilde{\varepsilon }}_{12}{}^{p}=\frac{\varepsilon_{12}{}^{pl}}{1-d_{12}}$$

where $\varepsilon_{12}$ is the total shear strain; $\varepsilon_{12}^{el}$ is the elastic shear strain, and $\varepsilon_{12}^{pl}$ is the plastic shear strain.

In the plane stress model, it is assumed that there is only plastic strain in the shear strain, and the yield function is shown as follows:

(19) $$F={\tilde{\tau }}_{12}-C{\left(\varepsilon_{12}{}^{pl}\right)}^{P}-{\tilde{\tau }}_{y0}$$

where ${\tilde{\tau }}_{y0}$ is the initial threshold of plastic strain, and *C* and *P* are the parameters in the hardening function.

The initial shear damage in the model is judged by the maximum stress criterion, which is expressed as follows:

(20) $$\phi_{12}=\frac{{\tilde{\tau }}_{12}}{S}$$

where $\phi_{12}$ is the shear failure factor; ${\tilde{\tau }}_{12}$ is the equivalent shear stress, and *S* is the initial shear damage stress.

The material shear damage variable adopts a decay method based on experimental data fitting. By comparing experimental data, the logarithm of effective shear stress shows a linear relationship with the shear damage variable, where the initial shear damage stress S can be calculated from the damage variable and shear stress, while $d_{12}$ can be obtained from shear cyclic loading tests [28].

**Figure 3:**
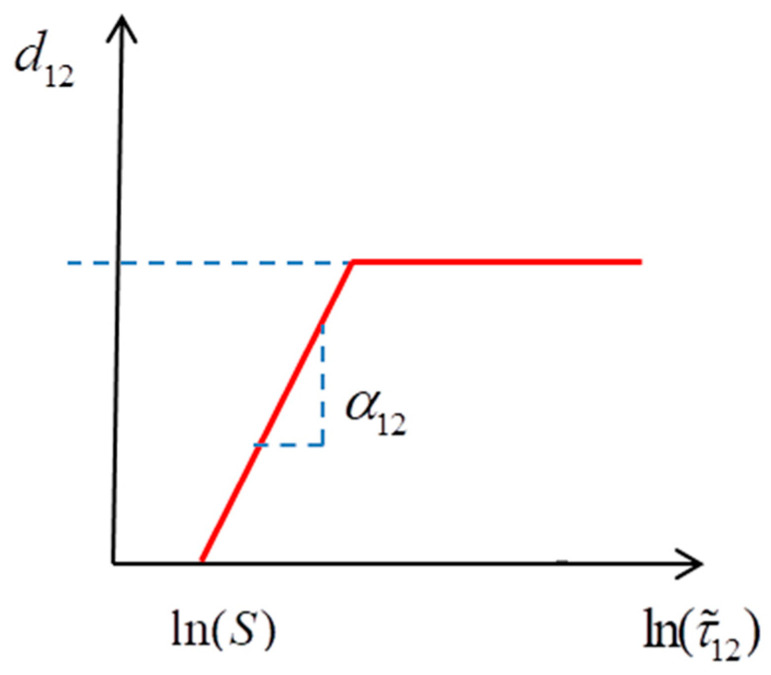
The relationship between the shear damage variable and shear stress [29,30].

The material shear damage variable $d_{12}$ adopts an attenuation method based on experimental data fitting. By comparing the experimental data, the logarithm of the effective shear stress $\ln({\tilde{\tau }}_{12})$ shows a linear relationship with the shear damage variable $d_{12}$ (shown in Figure 3) [29,30].

(21) $$d_{12}=\min\left(\alpha_{12}\ln\left(r_{12}\right),d_{12}^{\max}\right)$$

where $\alpha_{12}$ is the slope of the relationship curve between the logarithm and shear damage variable $d_{12}$ ($\alpha_{12}>0$); $d_{12}^{\max}$ is the maximum shear damage ($d_{12}^{\max}\leq 1$), and $r_{12}$ is the shear damage threshold ($r_{12}=\max_{\tau ⩽t}\phi_{12}(\tau )$).

#### 2.3.5. Delamination Failure Criteria

Considering the damage mode of the adhesive layer is caused by elastic brittle failure, the bilinear cohesive interface element damage constitutive model is selected for the adhesive layer. It is linear elastic before the initial damage occurs. After the adhesive layer element is damaged, the stress–strain of the material is [31,32]

(22) $$\left\{\left.\begin{array}{c} \sigma_{n}\\ \sigma_{s}\\ \sigma_{t} \end{array}\right\}\right.=(1-D)\left\{\left.\begin{array}{ccc} K_{nn} & & \\ & K_{ss} & \\ & & K_{tt} \end{array}\right\}\right.\left\{\left.\begin{array}{c} \delta_{n}\\ \delta_{s}\\ \delta_{t} \end{array}\right\}\right.$$

where *σ_n_* is the normal traction stress, and *σ_s_* and *σ_t_* are the shear stresses. *K_ii_* is the elastic stiffness coefficient; *δ_i_* is the stress of each direction, and *D* is the damage state variable.

The quadratic nominal stress criterion is adopted to judge the initiation damage [31,32]:

(23) $${\left(\frac{\sigma_{n}}{N_{\max}}\right)}^{2}+{\left(\frac{\sigma_{s}}{S_{\max}}\right)}^{2}+{\left(\frac{\sigma_{t}}{T_{\max}}\right)}^{2}=1$$

where *N*_max_, *S*_max_, and *T*_max_ are the strengths of the initial damage in each direction.

The damage propagation behavior of the adhesive layer is described by the Benzeggagh–Kenane (B–K) fracture criterion model [31,32]:

(24) $$G_{IC}+(G_{IIC}-G_{IC}){\left(\frac{G_{Shear}}{G_{T}}\right)}^{\eta }=G_{C}$$

(25) $$G_{T}=G_{I}+G_{Shear}$$

(26) $$G_{Shear}=G_{II}+G_{III}$$

where *G_IC_* and *G_IIC_* are the fracture toughnesses of types I and II; *G_I_*, *G_II_*, and *G_III_* are the strain energy release rates; *G_Shear_* is the shear strain energy release rate, and *η* is the material constant.

#### 2.3.6. Reliability Verification

Refer to the open-hole compression test data of thermoplastic composite materials to verify the effectiveness of the CF/PEEK composite material damage model in this part. The carbon fiber-reinforced thermoplastic composite material is an AS4D/PEEK prepreg. The mechanical performance indicators are shown in Table 5; the basic mechanical properties in the table are shown in the Refs. [21,33,34]; the fracture performance parameters are shown in the Refs. [21,35], and the parameters related to shear nonlinearity and damage definition are shown in the Ref. [35]. The various fitting parameters were calculated using the Iosipescu in-plane shear cyclic loading and unloading tests.

**Figure 4:**
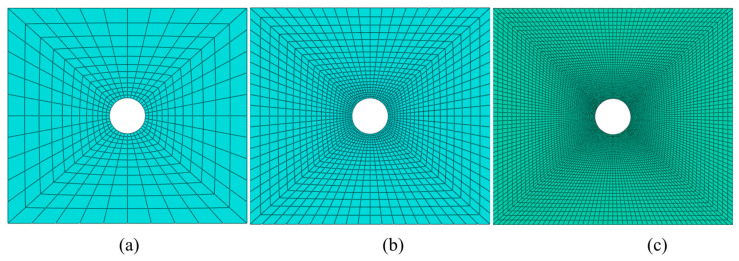
The compression calculation model for the laminated perforated structure. (**a**) Mesh size 0.5 mm. (**b**) Mesh size 0.25 mm. (**c**) Mesh size 0.15 mm.

Based on the geometric dimensions of the perforated compression test specimen in Section 2, a compression calculation model for the laminated, perforated structure was established for numerical calculation using the Abaqus Standard/Explicit model (DS SIMULIA, Providence, RI, USA). The stacking sequence is [45/0/–45/90/45/0/–45/90/45/0/–45/90/45/0/–45/90]s. The finite element model and boundary conditions of the laminated plate are shown in Figure 4. The calculation model includes 32 laminate layers and 31 interface cohesion layers. The interface cohesion layer is inserted between two layers, which is described using the cohesive zone model (CZM). The element type of laminate layers is C3D8R (an eight-node linear brick, reduced integration, hourglass control). The element type COH3D8 (an eight-node, three-dimensional cohesive element) is used to simulate the interface cohesion layer. Generally, when conducting numerical calculations, the smaller the unit size, the higher the accuracy, but the higher the calculation cost. Three models are established with the hole-edge mesh sizes of 0.15 mm, 0.25 mm, and 0.5 mm, respectively (shown in Figure 4), to verify the impact of mesh size on the analysis results. The properties of cohesion used in the calculation model are referred to in Refs. [31,32] (shown in Table 6).

**Figure 5:**
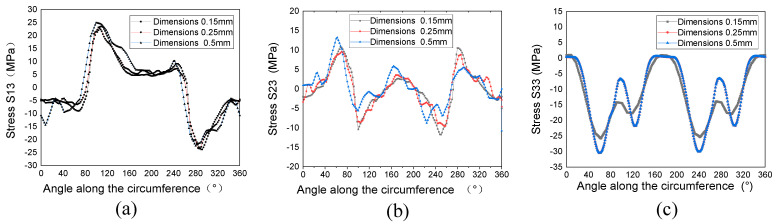
The interlaminar stress distribution along the hole edge at the interface +45/0 interface under compressive load. (**a**) Interlaminar shear stress S_13_. (**b**) Interlaminar shear stress S_23_. (**c**) Stress S_33_.

Figure 5 shows the interlaminar stress distribution along the hole edge at the interface +45/0 interface, obtained from the different grid sizes under compressive load. The stress distribution forms along the circumferential direction of the hole edge for the three grid sizes are basically consistent. From Figure 5a,b, it can be seen that the larger values of interlaminar shear stress S_13_ are located around 120° and 300°, and the larger values of S_23_ are located around 60°, 100°, 150°, 240°, and 290°, where shear failure is prone to occur. From Figure 5c, it can be seen that there is a significant interlaminar compressive stress S_33_ at the 60° and 240° attachments, which is prone to adhesive layer detachment failure. The analysis results have good consistency with the analysis results in Refs. [21,36].

**Figure 6:**
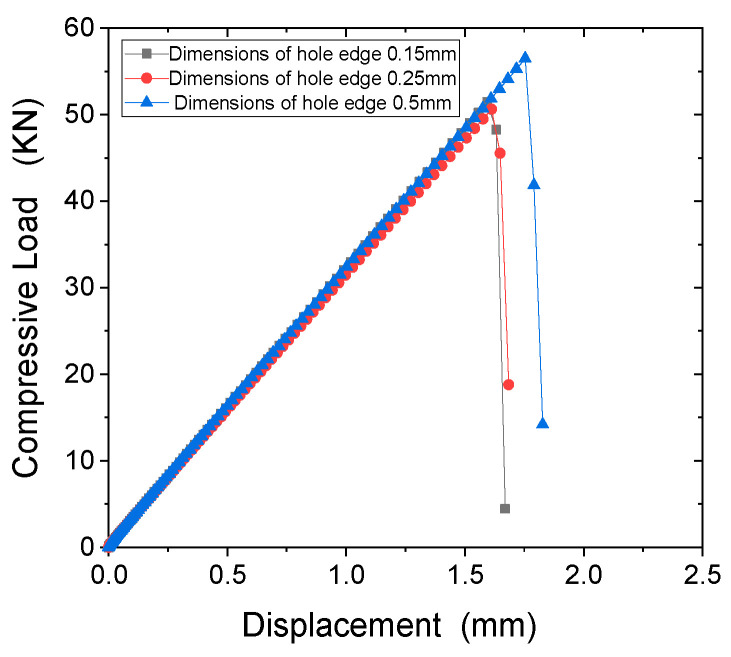
Compression load-displacement curve under different grid sizes under compression load.

The load-displacement curves of three sizes under compressive load are shown in Figure 6. From this Figure, it can be seen that the load-displacement curves and stress distribution along the axial direction of the hole edge are basically consistent for units with sizes of 0.15 mm, 0.25 mm, and 0.5 mm. The stress levels of 0.1 mm and 0.25 mm are similar, with an error of 1.53%. The stress levels of units with sizes of 0.5 mm are relatively large, with an error of 8.95%. Therefore, in the subsequent numerical calculations, a 0.25 mm element was used for this analysis.

## 3. Results and Discussion

Firstly, from a macro perspective, compare the differences in failure modes between thermoplastic composite materials and thermosetting composite materials in the open-hole tension, open-hole compression, and single-nail shear tests. To further understand the differences in micro-damage modes between thermosetting composite materials and thermoplastic composite materials, a comparative analysis was conducted on the micro-damage forms of fibers, matrices, and fiber/matrix interfaces between thermoplastic composite materials and thermosetting composite materials under compressive load based on Apeo-scanning electron microscope SEM images. Finally, the previous nonlinear damage prediction model with shear load was used to numerically simulate the opening performance of thermoplastic composite materials, revealing the impact of the damage initiation layer, damage initiation position, and stress distribution on the damage in the opening area of thermoplastic composite laminates under compressive load.

### 3.1. Compressive Behavior

#### 3.1.1. Experimental Results Comparison

**Figure 7:**
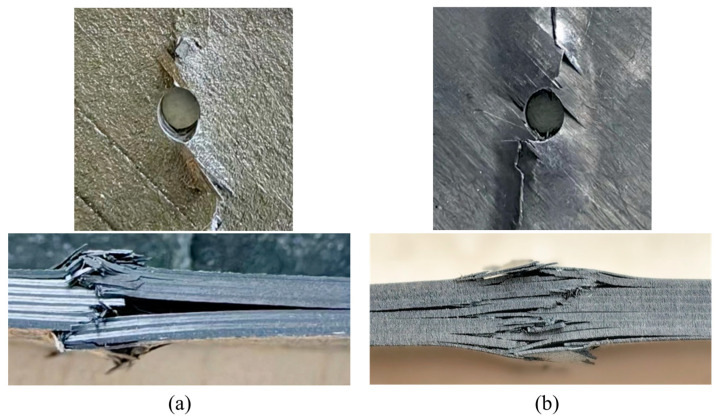
The typical macroscopic failure images of thermoplastic composite material TP and thermosetting composite material TS. (**a**) Thermosetting composite material TS. (**b**) Thermoplastic composite material TP.

Figure 7 shows the typical macroscopic failure images of thermoplastic composite material TP and thermosetting composite material TS under an open-hole compression. From the front face of the test piece, it can be seen that the fiber extrusion damage first appeared at the hole edge perpendicular to the load direction in the open-hole compression specimens. As the compression load increased, the fiber extrusion at the hole edge continued to increase and continued to expand laterally toward both sides of the test piece. From the side face of the test piece, it can be seen that both the thermoplastic composite material specimens and the thermosetting composite material specimens exhibit fiber extrusion and interlayer delamination, and the broken fibers are inserted into the opposite layered gap during the application of the compressive load, thereby promoting the continuous expansion of the delamination along the load direction and perpendicular to the load direction. In contrast, the delamination area of thermosetting composite materials is relatively concentrated, while the delamination distribution of thermoplastic composite materials is more uniform along the thickness direction.

**Figure 8:**
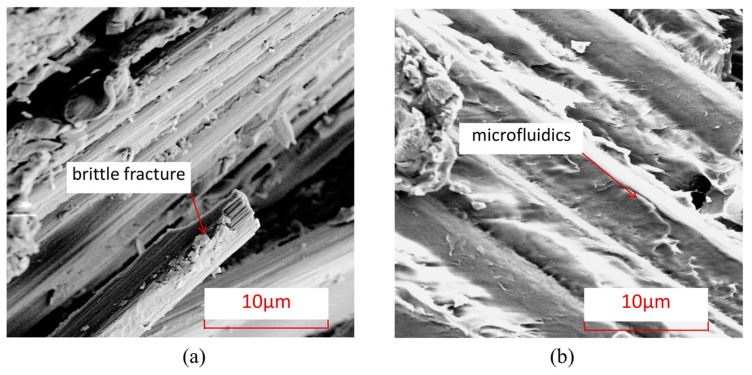
Electron microscopic scanning SEM images of fiber–resin interface damage under open-hole compression load. (**a**) Thermosetting composite material TS. (**b**) Thermoplastic composite material TP.

To further understand the microscopic damage forms of fibers and matrix in thermosetting and thermoplastic composites under an open-hole compression load, Figure 8 shows the damage electron microscopy scanning SEM images of the fiber/matrix interface in thermosetting composite material TS and thermoplastic composite material TP under an open-hole compression load. From this Figure, it can be seen that the fiber–matrix interface connection of thermoplastic composite material TP is stronger than that of the thermosetting composite material TS, and there is relatively little detachment of the fiber–resin interface. After compression failure, most of the fibers and matrix of thermoplastic composite material TP are still tightly connected.

**Figure 9:**
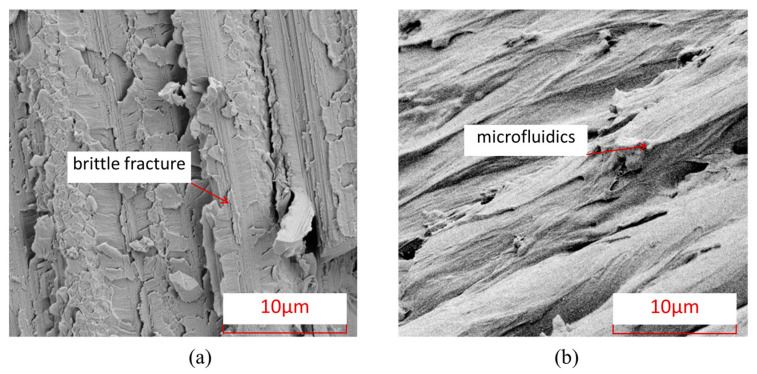
Electron microscopic scanning SEM images of thermosetting and thermoplastic resin damage under open-hole compression load. (**a**) Thermosetting composite material TS. (**b**) Thermoplastic composite material TP.

Figure 9 shows the characteristics of matrix damage in thermoplastic composite material TP and thermosetting composite material TS. From this Figure, it can be seen that the TP matrix exhibits ductile fracture characteristics, with obvious plastic deformation forming microfluidics in the matrix, while the TS matrix exhibits obvious brittle fracture characteristics, with most of the fracture areas of the matrix showing clear edge flake-like fractures.

#### 3.1.2. Failure Modes and Mechanisms

**Figure 10:**
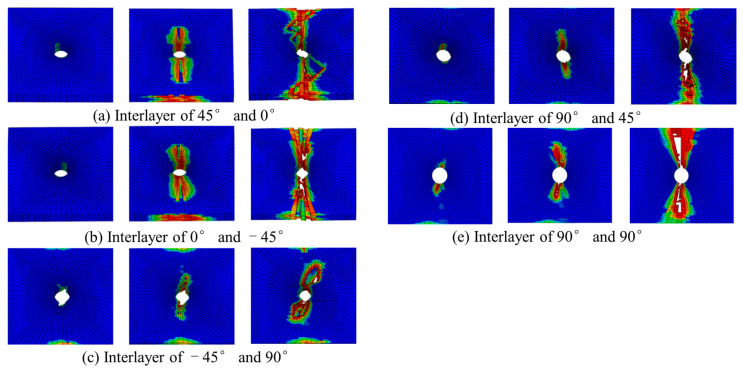
Evolution of adhesive layers damage in compression test.

The failure mode of composite materials is one of the important research objects. In this section, the failure mode and mechanisms are studied through finite element simulation. Figure 10 shows the delamination damage process. The delamination first occurs between 45° and 90° plies when the load reaches nearly 40% of the maximum load. Because the shear stress between 45° and 90° plies is the largest, delamination can easily occur. There is no delamination damage before the fiber extrusion fracture occurs. With the increase in the compression load, the damage to the matrix gradually increases. The delamination begins to occur from the hole edge. Then, it expands laterally and longitudinally. The delamination in the transverse region expands rapidly. Finally, the laminate fails completely in the transverse region caused by delamination.

**Figure 11:**
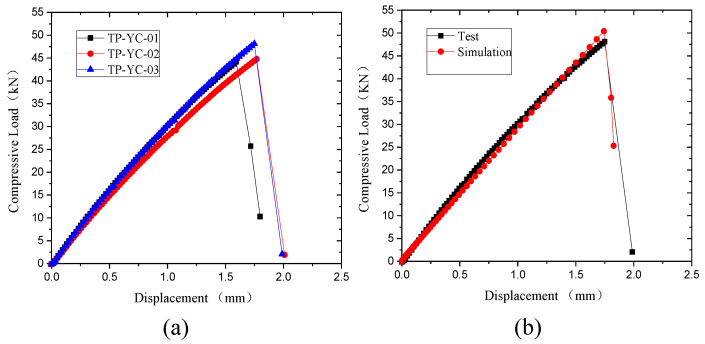
Compressive displacement-load curves. (**a**) Test results. (**b**) Comparison of test and simulation.

Figure 11a shows the load-displacement curve of the compression test. The compression process of the specimens is divided into three stages. The first stage is elastic, where the curve is a straight line. The second stage is damage extension after the early damage. The third stage is the failure stage, in which the fiber fracture extends to both sides, and the specimens are fractured. Figure 11b shows the comparison of test and simulation curves, which also proves the reliability of the simulation model.

**Figure 12:**
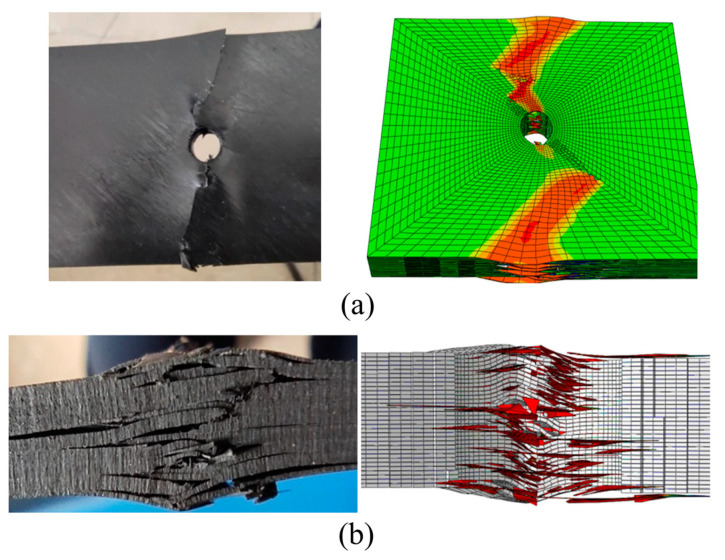
Experimental and simulation comparison of local failure under open-hole compression. (**a**) The front face of the test piece. (**b**) The side face of the test piece.

Figure 12 shows a comparison between experimental and simulation results of local failure of thermoplastic composite materials under open-hole compression. It can be seen from this Figure that the simulation and experiment have good consistency. Especially from the damage photos, it can be seen that under compressive load, fiber bundles break and extrude perpendicular to the load direction, and matrix cracking and interlayer delamination occur at the hole edge. Moreover, the delamination gradually expands along the loading direction and horizontally under the continuous compression of the fractured fibers, and ultimately, the fiber fracture and delamination damage extend to the short edge boundary, leading to the final fracture of the specimen.

### 3.2. Tensile Performance

#### 3.2.1. Experimental Results Comparison

**Figure 13:**
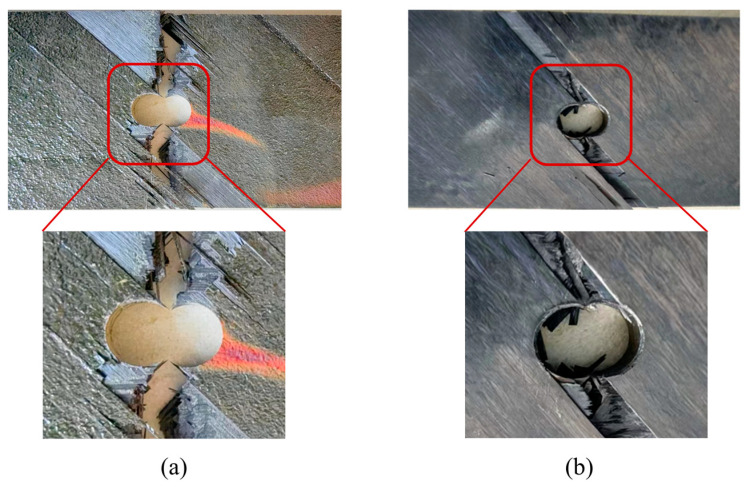
The typical macroscopic failure images of thermoplastic composite material TP and thermosetting composite material TS for tensile test. (**a**) Thermosetting composite material TS. (**b**) Thermoplastic composite material TP.

Figure 13 shows typical macroscopic failure images of thermoplastic composite material TP and thermosetting composite material TS under open-hole tension in the front plane and side planes. From the front face of the test piece in Figure 13a,b, it can be seen that the two materials’ open-hole tensile specimens first showed the fiber pull-out damage at the hole edge perpendicular to the load direction due to the fact that the tensile load of the 90° ply is mainly borne by the matrix, which has poor tensile capacity and separation between fiber bundles under tensile load. Subsequently, the shear load between the fiber bundles in the 45° ply also continued to increase, and there was a separation between the fiber bundles in the 45° ply. As the tensile load increased, the fibers in the 45° and 0° ply began to break and pull out and continued to expand laterally toward both sides of the test piece.

**Figure 14:**
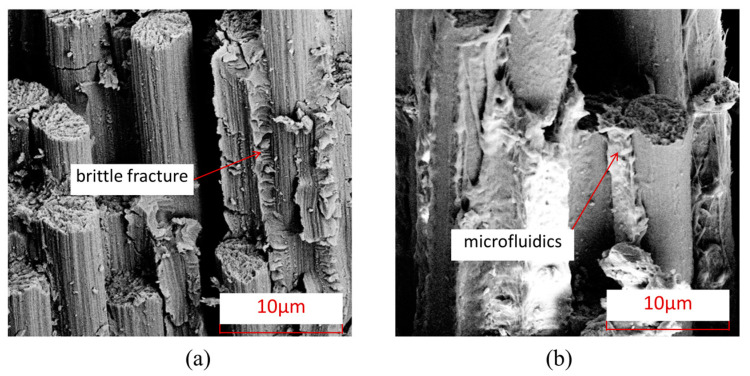
Electron microscopic scanning SEM images of fiber–resin interface damage under tensile load. (**a**) Thermosetting composite material TS. (**b**) Thermoplastic composite material TP.

**Figure 15:**
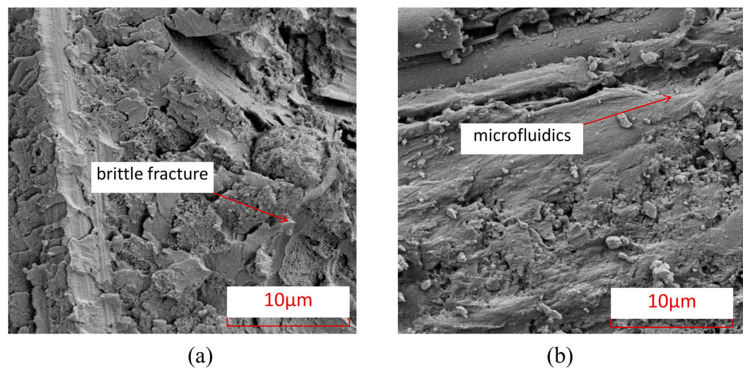
Electron microscopic scanning SEM images of thermosetting and thermoplastic resin damage under tensile load. (**a**) Thermosetting composite material TS; (**b**) Thermoplastic composite material TP.

To further understand the microscopic damage forms of fibers and matrix in thermosetting and thermoplastic composites under open-hole tensile load, Figure 14 presents SEM images of the damage at the interface between fibers and matrix in thermosetting composite material TS and thermoplastic composite material TP under open-hole tensile load. From this Figure, it can be seen that the interface connection between the fibers and the matrix of thermoplastic composite material TP is stronger than that between the matrix and fibers of thermosetting composite material TS, and there is relatively little detachment of the fiber–resin interface. After tensile failure, most of the fibers and matrix of TP are still tightly connected. Figure 15 shows the characteristics of matrix damage in thermoplastic composite material TP and thermosetting composite material TS. From this Figure, it can be seen that the TP matrix exhibits ductile fracture characteristics, with obvious plastic deformation forming microfluidics in the matrix, while the TS matrix exhibits obvious brittle fracture characteristics, with most of the fracture areas of the matrix showing clear edge flake-like fractures.

#### 3.2.2. Failure Modes and Mechanisms

**Figure 16:**
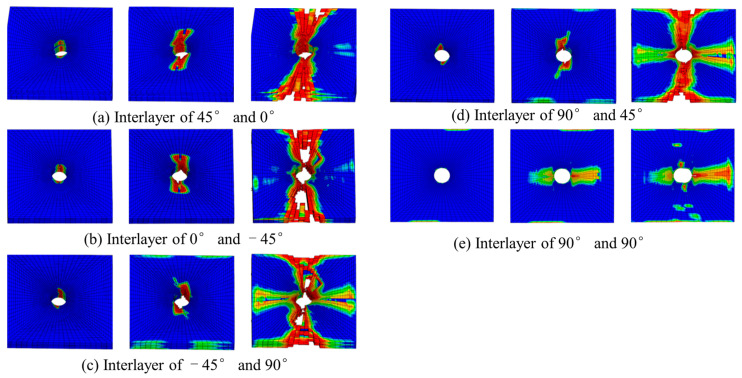
Damage process of adhesive layers under tensile load.

Figure 16 shows the damaging process of delamination. Different from the deformation process under compressive load, delamination damage occurs before the fiber bundle is damaged. With the increase in tensile load, the interfacial delamination between fiber and matrix intensifies. The matrix microcracks expand and pass through the single layer while the fiber bundles are pulled out and fractured. The laminate is completely delaminated in the transverse region and fails.

**Figure 17:**
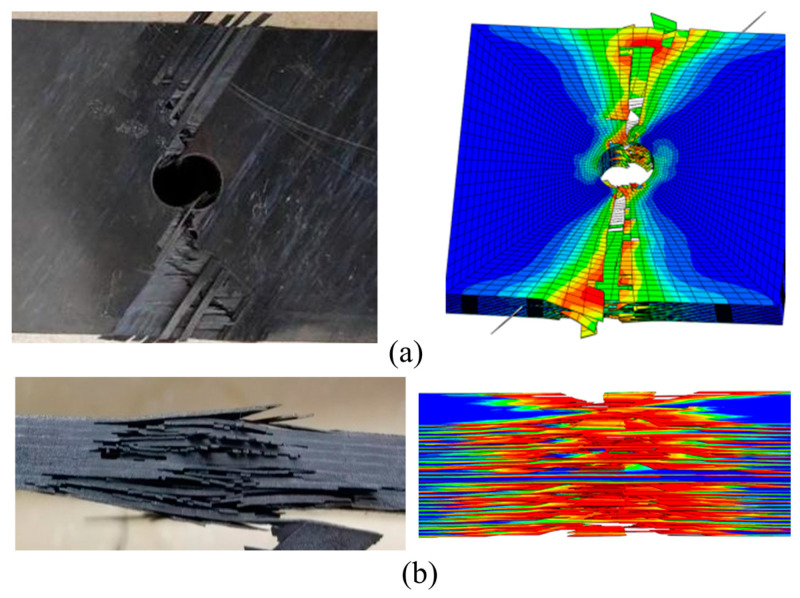
Tensile performances of the open-hole thermoplastic composites. (**a**) Damage details of the front view. (**b**) Damage details of the side view.

Figure 17 shows the typical failure modes of open-hole thermoplastic composite specimens with tensile load. The deformation process is observed. Firstly, the hole causes the stress concentration, which separates the interface between fiber and matrix. Then, it forms microcracks, which are manifested in fiber pull-out. Subsequently, the number of fibers pulled out near the hole increases. What is more, the fiber fracture increases and expands to both sides of the specimen. Finally, matrix cracks appear and expand, which form a visible delamination region.

**Figure 18:**
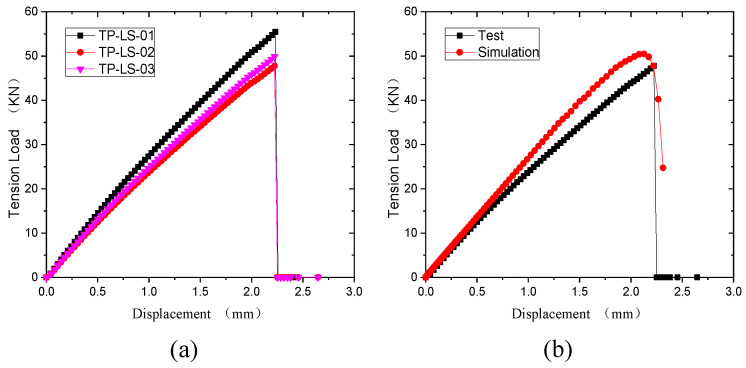
Tensile displacement-load curves of open-hole thermoplastic composites. (**a**) Experimental curves. (**b**) Results of test and simulation.

Figure 18 shows the displacement-load curves of the open-hole thermoplastic composite specimens under tensile load. It can be seen in Figure 18a that the tensile process is divided into three stages. The first stage is the elastic stage, where the load-displacement curve is a straight line. The second stage is the damage extension stage after early damage appears. In the third stage, the fiber fractures and is pulled out. The cracks extend to both sides laterally, so the laminate fractures. The comparison of the experimental and simulated curves is shown in Figure 18b. They match each other well, which proves the reliability of the simulation results.

### 3.3. Bearing Property

#### 3.3.1. Deformation Processes

**Figure 19:**
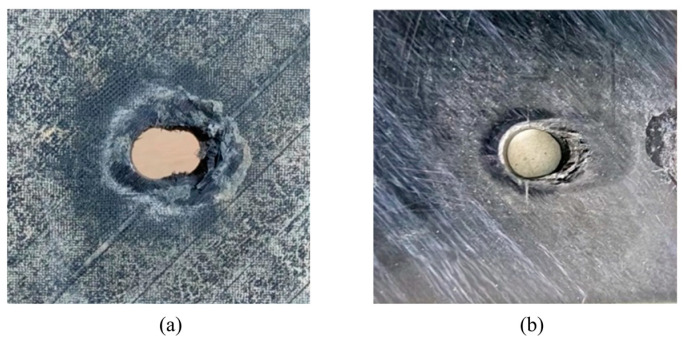
The typical macroscopic failure images of thermoplastic composite material TP and thermosetting composite material TS for bearing test. (**a**) Thermosetting composite material TS. (**b**) Thermoplastic composite material TP.

Figure 19 shows typical macroscopic failure images of thermoplastic composite material TP and thermosetting composite material TS under a single-shear compression load. From the plane specimens, which are shown in Figure 19a,b, it can be seen that the single-shear compression specimens of both materials exhibit compression failure in the direction of the end of the test piece. Thermoplastic composite materials mainly exhibit plastic flow in the matrix near the hole in contact with the bolt rod, and the fibers bend and break with numerical plastic deformation. However, thermosetting composite materials undergo greater deformation along the load direction, with more failure forms such as fiber and matrix separation, interlayer delamination, and matrix crushing appearing at the hole edge in contact with the bolt rod. In addition, thermoplastic composite materials and thermosetting composite materials exhibit small bracket-shaped matrix extrusion fractures and cracks in the contact area between the bolt cap and the hole edge in the load direction. As the extrusion load increases, the fracture area of the hole edge matrix increases, resulting in the microbending of fibers and interlayer delamination. When the damage continues to accumulate to a certain extent, local shear failure occurs in the test piece.

**Figure 20:**
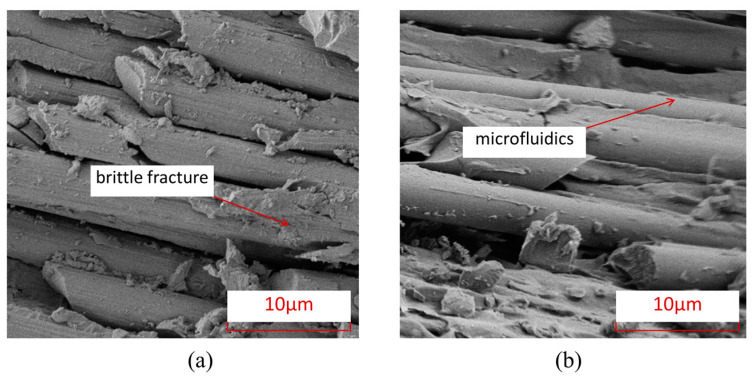
Electron microscopic scanning SEM images of fiber–resin interface damage under bolt-bearing load. (**a**) Thermosetting composite material TS. (**b**) Thermoplastic composite material TP.

**Figure 21:**
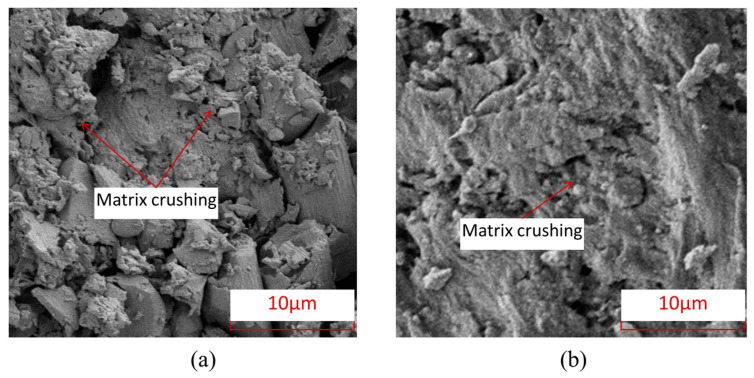
Electron microscopic scanning SEM images of thermosetting and thermoplastic resin damage under bolt-bearing load. (**a**) Thermosetting composite material TS. (**b**) Thermoplastic composite material TP.

Figure 20 shows the damage electron microscope scanning SEM images of the fibers and interfaces at the hole edge where the thermosetting composite material TS and thermoplastic composite material TP come into contact with the bolt rod under single-shear compression load. From this Figure, it can be seen that the interface connection between the fibers and the matrix of thermoplastic composite material TP is stronger than that between the matrix and fibers of thermosetting composite material TS, and there is relatively little detachment of the fiber–resin interface. After tensile failure, most of the fibers and matrix of TP are still tightly connected. Figure 21 shows the characteristics of matrix damage in the connection area between thermoplastic composite material TP and thermosetting composite material TS and the bolt rod. From this Figure, it can be seen that the TP matrix exhibits ductile fracture characteristics, with obvious plastic deformation formed by microflow and less obvious separation between the matrix and fibers. On the other hand, the TS matrix exhibits obvious brittle fracture characteristics, with both the matrix and fibers being arranged and crushed, and most of the fracture areas of the matrix are in the form of clear-edge flakes.

**Figure 22:**
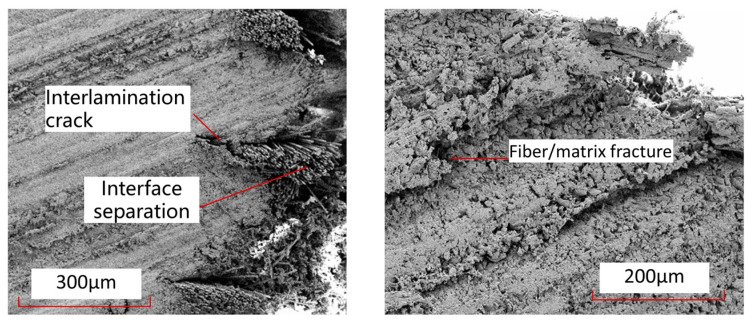
Local damage images of the contact area between the bolt rod and the nail hole in the single-shear extrusion of thermosetting composite materials.

**Figure 23:**
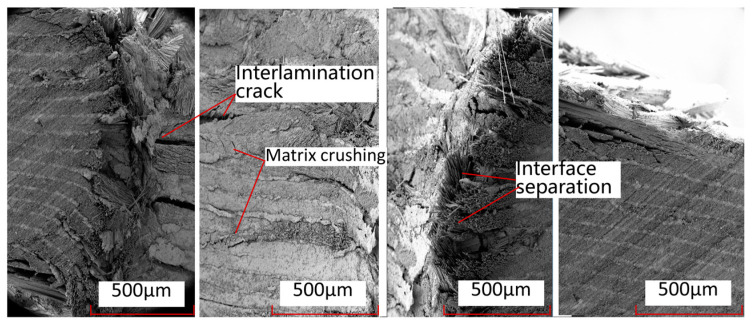
Local damage images of the contact area between the bolt rod and the nail hole in the single-shear extrusion of thermoplastic composite materials.

Figure 22 shows the local details of the rivet holes in the single-shear extrusion of thermosetting composite materials. From this Figure, it can be seen that the main damage modes in the middle part of the contact with the bolt rod are local fiber fracture, matrix crushing, matrix cracks, and interlayer delamination. The main damage modes in the edge part of the contact with the bolt rod are fiber–matrix interface separation, fiber fracture, interlayer delamination, and other damage modes. Figure 23 shows the local damage form at the connection between the nail hole and the bolt rod in thermoplastic composite laminates. From this Figure, it can be seen that there is a large range of fiber–matrix interface analysis, interlayer cracks, and local crushing of the matrix/fiber in the area near the nail hole to the surface of the laminates, but there is less damage in the upper and lower surface areas far away from the laminates.

**Figure 24:**
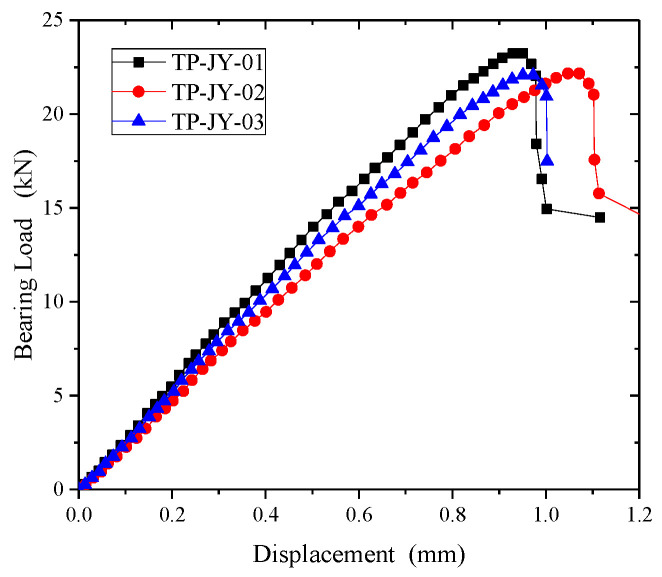
Bearing displacement-load curves of open-hole thermoplastic composites.

Figure 24 shows the bearing load-displacement curves of the open-hole thermoplastic composite specimens. The curves can be divided into three stages in the process of bearing. The first stage is the elastic stage. In the second stage, the microcrack of the matrix caused by the compressive stress appears near the hole, which leads to the matrix crushing and cracking. The sound of the matrix cracking can be heard in the test, and the slope of the curve decreases slightly. Matrix damage accumulates to the maximum during the third stage. The interface between matrix and fiber is separated, which promotes the instability buckling and the fracture damage of fiber. A loud noise occurs during the test. The damage around the extrusion hole expands continuously until permanent oblong damage occurs.

#### 3.3.2. Failure Modes and Mechanisms

**Figure 25:**
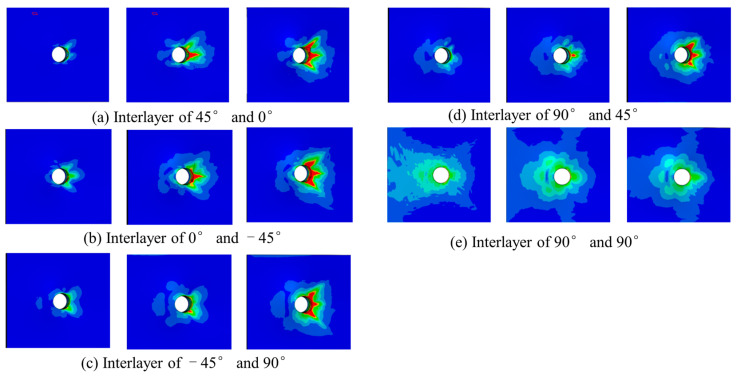
Evolution of adhesive layers damage in bearing test.

Figure 25 shows the delamination damage process. It first occurs between the 45° and 0° plies where the bolt head and bolt cap contact. The delamination damage occurs when the bearing load is nearly 40% of the maximum load. With the increase in the load, the delamination extends to the end area in 0°, 45°, and −45° directions.

**Figure 26:**
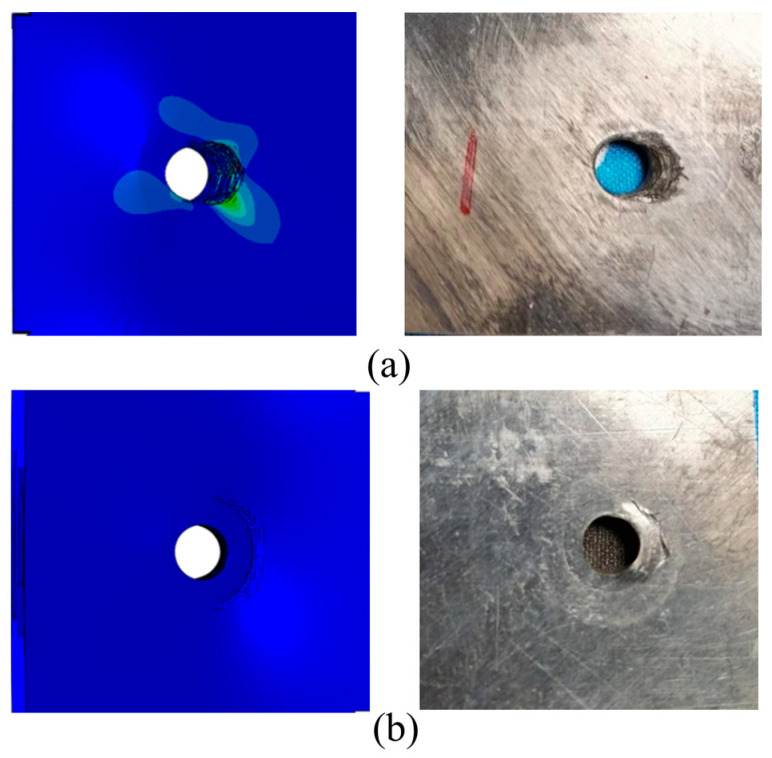
Comparison diagram of simulation tests for stress at bolt holes during single-shear extrusion. (**a**) Contact surface of left and right panels. (**b**) Contact surface between plate and bolt head.

**Figure 27:**
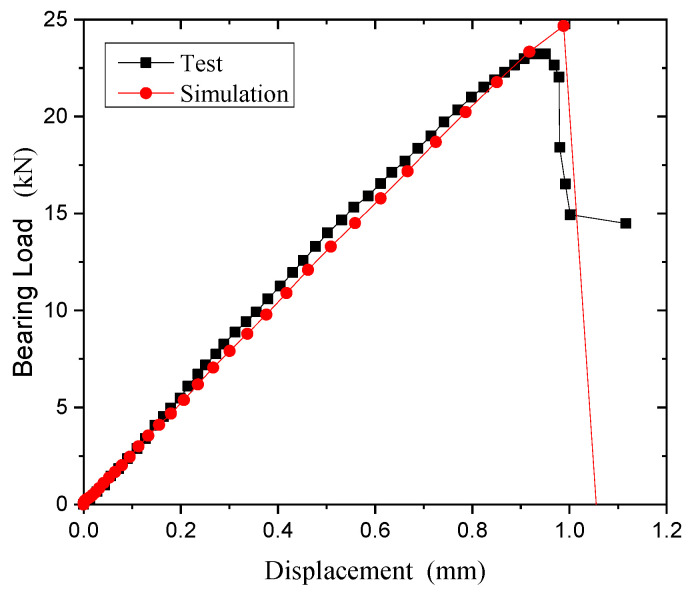
Comparison of simulation experiments on load-displacement curve of single-shear extrusion.

Figure 26 shows the Mises stress and experimental failure diagram at the bolt hole of single-shear extrusion. Figure 27 shows the comparison of the load-displacement curves between the experimental and simulated results under the bearing load. Figure 26a shows that there is a serious stress concentration phenomenon near the end hole wall on the contact side of the left and right composite laminates. The extrusion zone near the end hole wall first exhibits small bracket-shaped matrix compression fractures and cracks in the contact area between the bolt and the hole edge in the load direction. As the extrusion load increases, the matrix compression fracture area near the hole edge increases, resulting in microbending of fibers and interlayer delamination. When the damage continues to accumulate to a certain extent, the test piece undergoes local fragmented shear failure. At the contact surface between the plate and the bolt head in Figure 26b, the stress concentration is relatively weak, and there is a small compression failure in the outer area of the bolt head. The failure modes of the experiment and analysis have a good consistency.

## 4. Conclusions

(a) From a macro perspective, both thermoplastic composite material TP and thermosetting composite material TS exhibit fiber extrusion and interlayer delamination, and the broken fibers interpenetrate into the opposite layered gap, thereby promoting the continuous expansion of delamination along and perpendicular to the load direction. Compared to thermosetting composite materials, the delamination area is relatively concentrated, while TP delamination in thermoplastic composite materials is more evenly distributed along the thickness direction;
(b) From a macro perspective, both thermoplastic composite material specimens and thermosetting composite material specimens exhibit damage phenomena such as fiber bundle separation, fracture, pull-out, and interlayer delamination during the application of tensile load. The delamination area of thermosetting composite material is relatively concentrated, while the distribution of thermoplastic composite material delamination along the thickness direction is relatively uniform;
(c) The single-shear compression specimens of both materials showed compression failure in the direction of the end of the test piece. Thermoplastic composite materials mainly exhibit plastic flow in the matrix near the hole in contact with the bolt rod, and the fibers bend and break with numerical plastic deformation. However, thermosetting composite materials undergo greater deformation along the load direction, with more failure forms such as fiber and matrix separation, interlayer delamination, and matrix crushing appearing at the hole edge in contact with the bolt rod. In addition, thermoplastic composite materials and thermosetting composite materials exhibit small bracket-shaped matrix extrusion fractures and cracks in the contact area between the bolt cap and the hole edge in the load direction. As the extrusion load increases, the fracture area of the hole edge matrix increases, resulting in the microbending of fibers and interlayer delamination. When the damage continues to accumulate to a certain extent, local shear failure occurs in the test piece;
(d) From a microscopic perspective, the fiber–matrix interface connection of thermoplastic composite material TP is stronger than that of thermosetting composite material TS, and there is relatively little detachment of the fiber–resin interface. After compression failure, most of the fibers and matrix of thermoplastic composite material TP are still tightly connected. The thermoplastic matrix TP exhibits ductile fracture characteristics, with obvious plastic deformation forming microfluidics, while the thermosetting matrix TS exhibits obvious brittle fracture characteristics, with most of the fracture areas of the matrix showing clear edge sheet-like fractures.

## Figures and Tables

**Table 1:** Typical mechanical properties of carbon fibers.

	Tension Modulus (GPa)	Ultimate Tension Strength (MPa)	Elongation (%)	Manufacturer
AS4D	231	4347	1.88	Hexcel
CCF300	230	4210	1.78	Toray

**Table 2:** Mechanical parameters of AS4D/PEEK thermoplastic composites.

Density (MPa)	Poisson’s Ratio	Elastic Modulus (GPa)			Share Modulus (GPa)			Tensile Strength (MPa)			Compression Strength (MPa)			Shear Strength (MPa)		
ρ	*v*	E_1_	E_2_	E_3_	G_12_	G_13_	$G_{23}$	*σ_t_*_1_	*σ_t_*_2_	*σ_t_*_3_	*σ_c_*_1_	*σ_c_*_2_	*σ_c_*_3_	*τ*_12_	*τ*_13_	*τ*_23_
1580	0.3	130	9.3	9.3	4.1	3.85	3.85	1673	68	68	1436	257	257	136	86	121

**Table 3:** Mechanical parameters of CCF300/Epoxy thermosetting composites.

Density (MPa)	Poisson’s Ratio	Elastic Modulus (GPa)			Share Modulus (GPa)			Tensile Strength (MPa)			Compression Strength (MPa)			Shear Strength (MPa)		
ρ	*v*	E_1_	E_2_	E_3_	G_12_	G_13_	G_23_	*σ_t_*_1_	*σ_t_*_2_	*σ_t_*_3_	*σ_c_*_1_	*σ_c_*_2_	*σ_c_*_3_	*τ*_12_	*τ*_13_	*τ*_23_
1600	0.3	130	9.3	9.3	4.1	3.85	3.85	1673	68	68	1436	257	257	136	86	121

**Table 4:** Typical mechanical properties of matrix.

	Tension Modulus (MPa)	Tension Strength (MPa)	Elongation (%)	Fracture Toughness (J/mm)
PEEK	3.8	94	50	2000
Epoxy	3.58	105	1.9	420

**Table 5:** Fracture performance parameters and shear nonlinearity parameters of AS4D/PEEK thermoplastic composites.

Density ($kg/m^{3}$)	Poisson’s Ratio	Elastic Modulus (GPa)			Share Modulus (GPa)			Tensile Strength (MPa)		
ρ	v	$E_{1}$	$E_{2}$	$E_{3}$	$G_{12}$	$G_{13}$	$G_{23}$	*σ_t_*_1_	*σ_t_*_2_	*σ_t_*_3_
1580	0.3	130	9.7	9.7	5.2	3.94	3.94	2280	69	69
**Compression** **Strength (** MPa **)**			**Shear** **Strength (** MPa **)**			**Longitudinal Fracture** **Toughness (KJ/m^2^)**		**Transverse Fracture** **Toughness (KJ/m^2^)**		
***σ_c_*** **_1_**	***σ_c_*** **_2_**	***σ_c_*** **_3_**	***τ*** **_12_**	***τ*** **_13_**	***τ*** **_23_**	$G_{f}^{+1}$	$G_{f}^{-1}$	$G_{f}^{+2}$	$G_{f}^{-2}$	
1300	208	208	152	94	94	90	82	0.52	1.61	
**Shear Damage** **Coefficient**		**Shear Damage Factor**		**Initial Yield Stress**		**Plastic Hardening** **Coefficient**		**Coefficient**		
$\alpha_{12}$		$d_{12}^{\max}$		$\tau_{y0}$		***C***		***p***		
176		0.67		25.2		182.52		0.2553		

**Table 6:** Mechanical parameters of cohesive.

Elastic Modulus (N/mm)			Fracture Toughness (N/mm)			Strength (MPa)		
$K_{nn}$	$K_{ss}$	$K_{tt}$	$G_{IC}$	$G_{IIC}$	$G_{IIIC}$	$\sigma_{n}$	$\sigma_{s}$	$\sigma_{t}$
2.207 × 10^10^	9.047 × 10^9^	9.047 × 10^9^	0.37	0.82	0.82	33	54	54

## Data Availability

The data presented in this study are available on request from the corresponding author.
